# Effect of the APOE Polymorphism and Age Trajectories of Physiological Variables on Mortality: Application of Genetic Stochastic Process Model of Aging

**DOI:** 10.6064/2012/568628

**Published:** 2012-07-02

**Authors:** Konstantin G. Arbeev, Svetlana V. Ukraintseva, Alexander M. Kulminski, Igor Akushevich, Liubov S. Arbeeva, Irina V. Culminskaya, Deqing Wu, Anatoliy I. Yashin

**Affiliations:** ^1^Center for Population Health and Aging, Duke University, P.O. Box 90408, Durham, NC 27708-0408, USA; ^2^Duke Cancer Institute, Duke University, Durham, NC 27710, USA

## Abstract

We evaluated effects of the APOE polymorphism (carriers versus noncarriers of the e4 allele) and age trajectories of total cholesterol (CH) and diastolic blood pressure (DBP) on mortality risk in the Framingham Heart Study (original cohort). We found that long-lived carriers and noncarriers have different average age trajectories and long-lived individuals have consistently higher levels and less steep declines at old ages compared to short-lived individuals. We applied the stochastic process model of aging aimed at joint analyses of genetic and nongenetic subsamples of longitudinal data and estimated different aging-related characteristics for carriers and noncarriers which otherwise cannot be evaluated from data. We found that such characteristics differ in carriers and noncarriers: (1) carriers have better adaptive capacity than noncarriers in case of CH, whereas for DBP the opposite situation is observed; (2) mean allostatic trajectories are higher in carriers and they differ from “optimal” trajectories minimizing mortality risk; (3) noncarriers have lower baseline mortality rates at younger ages but they increase faster than those for carriers resulting in intersection at the oldest ages. Such observations strongly indicate the presence of a genetic component in respective aging-related mechanisms. Such differences may contribute to patterns of allele- and sex-specific mortality rates.

## 1. Introduction

The apolipoprotein E (APOE) polymorphism is one of the most studied polymorphisms in humans. It has been extensively studied for its associations with various aging-related disorders such as cognitive impairment, Alzheimer's disease, atherosclerosis, stroke, diabetes, and cancer [1–5]. Its involvement in regulation of various aspects of aging has been discussed in the literature [6–8]. Nevertheless, the effect of APOE on survival evaluated in different longitudinal studies still remains contradictory (see [9–11], and references therein). 

Survival is a complex phenotype summarizing the contribution of different factors during the entire life course of an individual. Therefore, longitudinal studies on aging, health, and longevity collecting measurements of various physiological variables during a substantially long-time period, along with data on mortality and information on genetic markers, provide a valuable source of information for investigation of genetic contribution to the aging-related processes leading to an increase in the risk of death. However, longitudinal data typically contain limited information that can be directly associated with mechanisms of aging-related changes in human organisms, such as homeostatic regulation, allostatic adaptation, and stress resistance. The lack of available information limits empirical analyses of longitudinal data aimed at genetic analyses of such mechanisms. In such circumstances, mathematical modeling can help in joint analyses of age trajectories of physiological variables (which can reflect the influence of different external and internal processes during the individual's life) and data on mortality and genetic markers. Such studies can help investigate regularities in aging-related changes hidden in the age dynamics of physiological variables and evaluate the impact of genetic factors on corresponding underlying mechanisms leading to deterioration in health and death. The appropriate model for such analyses, the stochastic process model of aging, has been developed recently by authors of this paper [12–15]. The specific version of this model that incorporates genetic information was developed in [13]. We will denote this model here as the “genetic stochastic process model” (or “GenSPM”). This model incorporates several major concepts of aging including age-specific physiological norms, allostasis and allostatic load, stochasticity, and decline in stress resistance and adaptive capacity with age. The approach allows for evaluating all these characteristics in their mutual connection, even if respective aging-related mechanisms are not directly measured in data (which is typical for longitudinal data available to date). The model takes into account the dependence of age trajectories of physiological variables and hazard rates on genetic markers and permits evaluation of all these aging-related characteristics for carriers of different alleles (or genotypes). The model also combines data for individuals for whom genetic data were collected (“genetic subsample”) and for those without such information (“nongenetic subsample”). Similarly to the method combining genetic and nongenetic subsamples in analyses of longitudinal data on survival without the inclusion of measurements of physiological variables [16], the GenSPM substantially increases the accuracy of parameter estimates compared to the analyses of information from a genetic subsample alone [13].

In this study, we apply the GenSPM to the genetic subsample of the original cohort of the Framingham Heart Study (FHS) containing information on the APOE polymorphism (carriers and noncarriers of the e4 allele) and to data on mortality and longitudinal measurements of physiological variables (such as total cholesterol and diastolic blood pressure, which are available in the most of (or all) FHS exams) which are available for both genetic and nongenetic subsamples of the FHS. We evaluate and compare different aging-related characteristics for carriers and noncarriers of the APOE e4 allele which may jointly contribute to the patterns of the allele-specific mortality rates. 

## 2. Data and Methods

### 2.1. Framingham Heart Study (FHS) Data

The original FHS cohort consists of 5,209 respondents (nearly all are Caucasians, 46% male) aged 28–62 years at baseline and residing in Framingham, Massachusetts, between 1948 and 1951, and who had not yet developed overt symptoms of cardiovascular disease or suffered a heart attack or stroke [17, 18]. The study continues to the present with biennial examinations (30 exams to date; data from exams 1–26 were available for this study) that include detailed medical history, physical exams, and laboratory tests. Examination of participants, including an interview, physical examination, and laboratory tests, has been taken biennially. The original cohort has been followed for more than 60 years (information on about 55 years of followup was available for this study) for the occurrence of diseases (such as cardiovascular diseases and cancer) and death through surveillance of hospital admissions, death registries, and other available sources. In this study, we used data on the number of days since the date of exam 1 until the date of event (death) or censoring from the folowup dataset to calculate ages at death/censoring for participants of the original cohort. Longitudinal measurements of total cholesterol (denoted CH throughout the text) from exams 1–11, 13–15, 20 and 22–26 were used in this study. Measurements of diastolic blood pressure (DBP) were available in all 26 exams.

The dataset available for this study contained information on 5,079 participants of the original cohort (2,785 females; 2,294 males). We excluded from analyses individuals for whom measurements of physiological variables were not available in any exam. The resulting sample of 5,051 individuals (2,773 females; 2,278 males) with at least one measurement of CH was used in analyses of the genetic stochastic process model described below. All 5,079 individuals had at least one measurement of DBP; therefore, the entire sample was used in applications of the model to data on DBP. Individuals who did not die within two years (which is the average period between the exams in the original FHS cohort) since the last observation of a physiological variable were censored at respective ages, or at the latest ages for which information on their vital status was available, whichever were the earliest. 

APOE genotyping in the original FHS cohort was performed using DNA samples collected during the 19th examination (years 1986-1987) as described elsewhere (see, e.g., [19]). For the present study, data on the APOE polymorphism were available for 1,258 participants (802 females, 456 males) of the original FHS cohort. We refer to this subsample as the “APOE subsample” or “genetic subsample.” In the FHS APOE subsample, 277 individuals (183 females, 94 males) were carriers of the e4 allele (genotypes e2/e4, e3/e4, or e4/e4) and 981 individuals (619 females, 362 males) were noncarriers of that allele (genotypes e2/e2, e2/e3, or e3/e3). Survival data from the entire FHS original cohort were available for this study (as described above, for 5,051 individuals in analyses of CH data and 5,079 individuals in analyses of DBP data). We refer to this sample as the “combined APOE and non-APOE subsamples” or just the “entire FHS sample” to indicate that survival data were available for those with and without genetic information (for which we use the term “nongenetic subsample” or “non-APOE subsample”). 

### 2.2. Empirical Analyses of Age Dynamics of Physiological Variables in Carriers and Noncarriers of the APOE e4 Allele

We evaluated average age trajectories of physiological variables (CH and DBP) in long-lived female and male carriers and noncarriers of the e4 allele using pooled data on measurements from all FHS exams. Individuals were classified as long-lived if they survived until the age where the sex-specific survival functions reached 0.2 (which is about 90 years for females and 85 years for males in the FHS sample).

We also calculated average age trajectories of these physiological variables for female and male carriers and noncarriers of the e4 allele who survived until different ages. We separated females into three subgroups: the first includes those who survived until age 90 years (which is the same long-lived group described above) and the next two are those who died at ages 80–89 years and less than 80 years. Note that censored individuals were included in the first group but not in the second and third ones. Due to smaller sample sizes, we separated males into two groups: the long-lived group (who survived until age 85 years) and those who died at ages less than 85 years. Again, censored individuals were included in the first group but not in the second one.

### 2.3. Statistical Analyses: The Model Describing Age Dynamics of Physiological Variables and Mortality Risks in Carriers and Noncarriers of the APOE e4 Allele

We used the discrete time version of the genetic stochastic process model (GenSPM) [13]. Values of physiological variables (CH and DBP) were evaluated at one-year age intervals using a linear approximation of respective observations in the adjacent FHS exams. The model was applied to data on mortality in the combined APOE and non-APOE subsamples of the original FHS cohort. The details of the likelihood maximization procedure are given in [13]. Below, we provide specifications of the version of the GenSPM used in this study.

Let a discrete random variable *G* (*G* = 0, 1; *P*(*G* = 1) = *p*_1_) characterize the absence (*G* = 1) or presence (*G* = 0) of the APOE e4 allele in the genome of an individual. Let *Y*_*t*_ be the random process modeling the dynamics of a physiological variable (*t* is age). We assume that the evolution of *Y*_*t*_ depends on the presence or absence of the e4 allele in the genome and it may be described by the following stochastic differential equation with coefficients depending on the values of *G*:

(1) $$\begin{array}{c} dY_{t}=a\left(t,G\right)\left(Y_{t}-f_{1}\left(t,G\right)\right)dt+B\left(t,G\right)dW_{t}, \end{array}$$

with the initial condition *Y*_*t*_0__ ~ *N*(*f*_1_(*t*_0_, *G*), *σ*_0*G*_), *G* = 0, 1, where the parameters *σ*_0*G*_ are estimated from the data. Here *W*_*t*_ is a Wiener process independent of  *Y*_*t*_0__ and *G*. It describes external disturbances affecting these physiological variables and incorporates *stochasticity* into the model. The strength of disturbances is characterized by the diffusion coefficient *B*(*t*, *G*). The diffusion coefficient *B*(*t*, *G*) was modeled constant (*B*(*t*, *G*) = *σ*_1*G*_) in these applications. 

The function *f*_1_(*t*, *G*) introduces the notion of *allostasis* into the model and it may be referred to as the “mean allostatic trajectory.” This function describes the effect of allostatic adaptation [20], that is, this is the trajectory that a physiological variable is forced to follow by homeostatic forces in the presence of external disturbances described by the Wiener process *W*_*t*_. We used the quadratic function to model the mean allostatic trajectories *f*_1_(*t*, *G*): *f*_1_(*t*, *G*) = *a*_*f*_1__^*G*^ + *b*_*f*_1__^*G*^ *t* + *c*_*f*_1__^*G*^ *t* ^2^. The choice of the quadratic function for the mean allostatic trajectories comes from the empirical observations of the average trajectories of the physiological variables in the FHS, which generally have a quadratic form [21], although, of course, these average trajectories do not necessary have to follow *f*_1_(*t*, *G*).

The strength of homeostatic forces is characterized by the negative feedback coefficient *a*(*t*, *G*), larger values of this function correspond to a faster return of the trajectory of a physiological variable to the allostatically prescribed values *f*_1_(*t*, *G*). Therefore, the decline in the absolute value of this function with age represents the decline in the adaptive (homeostatic) capacity with age (“homeostenosis”) which has been shown to be an important characteristic of aging [22–25]. We used a linear approximation of the decline in the adaptive capacity with age, that is, the feedback coefficient *a*(*t*, *G*): *a*(*t*, *G*) = *a*_*Y*_^*G*^ + *b*_*Y*_^*G*^ *t* (with *a*_*Y*_ < 0 and *b*_*Y*_ ≥ 0). 

Different studies observed U- or J-shape of the mortality and morbidity risks as functions of various physiological variables [26–31]. Thus, it may be argued based on these observations that a quadratic function can model dependence of the risk on deviations of trajectories of a physiological variable *Y*_*t*_ from its “optimal” values [13, 14, 32–35]. Let the mortality rate conditional on *Y*_*t*_ and *G* be

(2) $$\begin{array}{c} \mu \left(t,Y_{t},G\right)=\mu_{0}\left(t,G\right)+{\left(Y_{t}-f\left(t,G\right)\right)}^{2}\mu_{1}\left(t,G\right). \end{array}$$

Here the function *μ*_0_(*t*, *G*) is the background (baseline) hazard characterizing the residual mortality rate, which would remain if physiological variables (*Y*_*t*_) follow their “optimal” trajectories, that is, coincide with the function *f*(*t*, *G*). Thus, *μ*_0_(*t*, *G*) is associated with death from factors other than those involved in the quadratic part and represented by *Y*_*t*_ (i.e., with unmeasured factors). We used the gamma-Gompertz (logistic) baseline hazards *μ*_0_(*t*, *G*): *μ*_0_(*t*, *G*) = *μ*_0_^0^(*t*, *G*)/(1 + *σ*_2*G*_^2^∫_0_^*t*^ *μ*_0_^0^(*u*, *G*)*du*), where *μ*_0_^0^(*t*, *G*) = *a*_*μ*_0__^*G*^ *e* ^*b*_*μ*_0__^*G*^*t*^. This choice for the baseline hazard takes into account the possibility of deceleration of mortality rate at the oldest old ages [36] which cannot be captured by the Gompertz curve.

The nonnegative multiplier *μ*_1_(*t*, *G*) in the quadratic part of the hazard characterizes sensitivity of the risk function (mortality rate) to deviations of a physiological variable from the “optimal” values *f*(*t*, *G*). This multiplier can be interpreted in terms of the “robustness,” or “vulnerability,” component of stress resistance. An increase of this function with age corresponds to narrowing U shape of the risk with age, that is, an organism becomes more vulnerable to deviations from the “optimal” values (because the same magnitude of deviations from the “optimal” trajectory result in a larger increase in the risk). Thus, an increase in *μ*_1_(*t*, *G*) with age corresponds to the decline in stress resistance which can be considered as a manifestation of the senescence process [37, 38]. We specified *μ*_1_(*t*, *G*) as a linear function of age: *μ*_1_(*t*, *G*) = *a*_*μ*_1__^*G*^ + *b*_*μ*_1__^*G*^ *t*.

**Figure 1:**
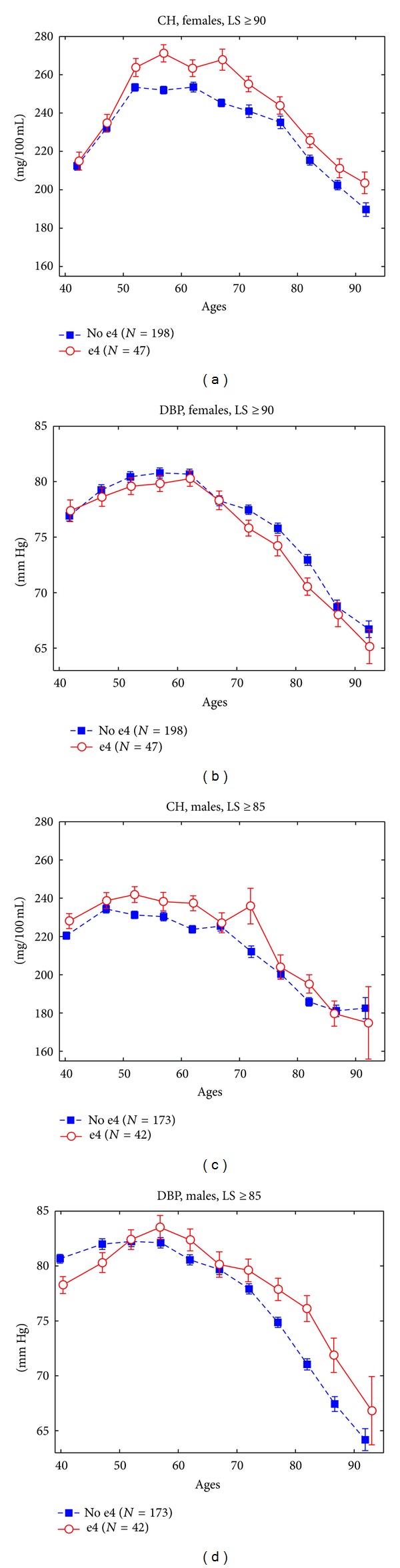
Average age trajectories (±S.E.) of total cholesterol (“CH”) and diastolic blood pressure (“DBP”) for long-lived female (life span (“LS”) ≥ 90 years) and male (LS ≥ 85 years) carriers (“e4”) and noncarriers (“No e4”) of the APOE e4 allele in the Framingham Heart Study (original cohort). “*N*” denotes the number of individuals.

**Figure 2:**
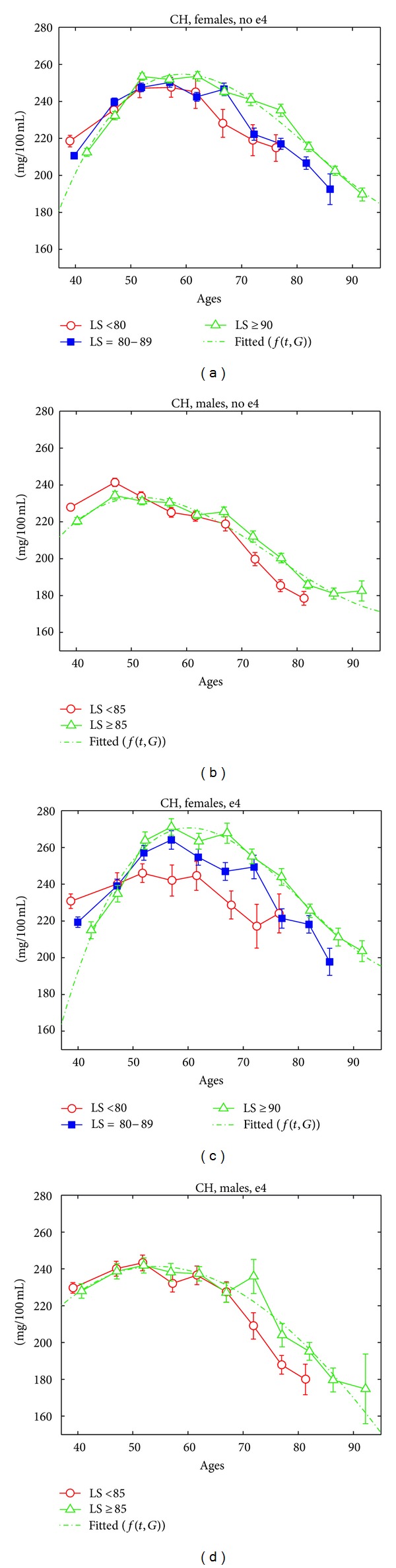
Average age trajectories (±s.e.) of total cholesterol (“CH”) for female and male carriers (“e4”) and noncarriers (“no e4”) of the APOE e4 allele who survived until different ages (“LS” denotes life span); *f*(*t*, *G*) are age trajectories for the long-lived groups fitted by cubic polynomials (used as physiological “norms” in the genetic stochastic process model, see the text). Data source: Framingham Heart Study (original cohort).

**Figure 3:**
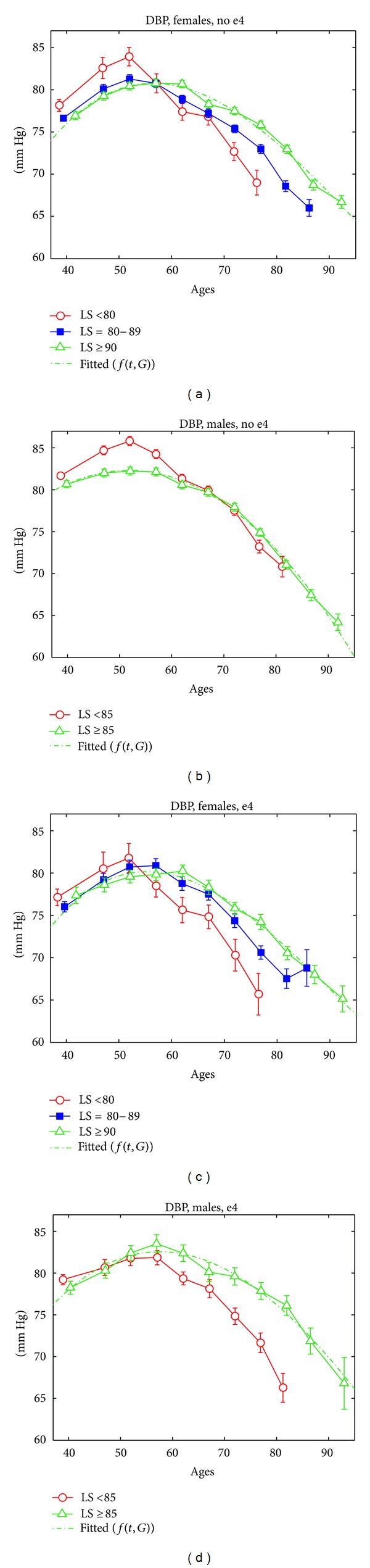
Average age trajectories (±s.e.) of diastolic blood pressure (“DBP”) for female and male carriers (“e4”) and noncarriers (“no e4”) of the APOE e4 allele who survived until different ages (“LS” denotes life span); *f*(*t*, *G*) are age trajectories for the long-lived groups fitted by cubic polynomials (used as physiological “norms” in the genetic stochastic process model, see the text). Data source: Framingham Heart Study (original cohort).

To represent the “optimal” trajectories *f*(*t*, *G*) in the model, we calculated the average age trajectories (in 5-year age groups, from ages 40–44 to 90+) of respective physiological variables in long-lived (life span ≥ 90 for females; life span ≥ 85 for males) female and male carriers and noncarriers of the APOE e4 allele. These empirical trajectories were then fitted by cubic polynomials and these fitted trajectories were used as the “optimal” trajectories *f*(*t*, *G*) in the model (see Figures 1–3).

Note that all parameters in the model depend on *G*. This allows for testing the hypotheses on the differences in aging-related characteristics (e.g., adaptive capacity, mean allostatic trajectories, etc.) between carriers and noncarriers of the e4 allele. Other hypotheses (e.g., on the decline in adaptive capacity with age, etc.) can also be tested. We tested all such hypotheses using the likelihood ratio test. For example, to test the null hypothesis about the equality of the adaptive capacity in carriers and noncarriers of the e4 allele, we estimated the likelihood function in the “general” model with separate *a*(*t*, *G*) in carriers and noncarriers and in the “restricted” model with *a*(*t*, 1) = *a*(*t*, 0) (all other functions except *a*(*t*, *G*) were specified similarly in both models), and then applied the likelihood ratio test. All statistical analyses of the GenSPM (the likelihood optimization and the statistical tests) have been performed using Optimization and Statistical Toolboxes in MATLAB R2010a. 

## 3. Results and Discussion

### 3.1. Empirical Analyses

We analyzed age trajectories of physiological variables (CH and DBP) in long-lived carriers and noncarriers of the APOE e4 allele (Figure 1). The figure shows that long-lived carriers and noncarriers of the e4 allele have different average age trajectories of CH and DBP. Long-lived female carriers and noncarriers have about the same level of CH at age 40 and about the same rate of increase at ages 40–50. However, at older ages, the level of CH is consistently higher in female carriers of the e4 allele and the rate of decline is almost the same in carriers and noncarriers at ages 75+. Long-lived male carriers and noncarriers of the e4 allele have generally lower levels of CH at ages 50+, compared to females, which is similar to the patterns observed in the entire sample [21]. Long-lived male carriers of the e4 allele, however, have higher levels of CH at ages until 85, compared to noncarriers of this allele. Differences between the trajectories of DBP in long-lived female carriers and noncarriers are less pronounced than those for CH. Nevertheless, starting with about the same level at age 40, long-lived female carriers of the e4 allele afterwards have a generally lower level of DBP, compared to long-lived noncarriers. Following the pattern in the entire sample [21], both long-lived male carriers and noncarriers have higher levels of DBP at younger ages (up to 65–70 years), compared to females. However, the age dynamics of DBP differs in long-lived male carriers and noncarriers of the e4 allele; carriers have lower values of DBP at ages until about 55 years and then their average level of DBP becomes higher than in noncarriers and the difference increases at the oldest ages.

We should note that the average trajectories of physiological variables in long-lived individuals shown in Figure 1 are not influenced by the effects of compositional changes in the sample due to attrition (mortality). In the total sample, compositional changes due to attrition may affect the averaging procedure and modify the sample means. It can happen because the levels and the age dynamics of physiological variables are related to the mortality risk (see, e.g., our recent studies with the FHS data [32, 35, 39]). Figures 2 and 3 illustrate that this is also true for carriers and noncarriers of the APOE e4 allele. We found that carriers and noncarriers of the APOE e4 allele with different life spans have different average age trajectories of CH (Figure 2) and DBP (Figure 3). Figures 2(a) and 2(c) show that the age trajectories of CH in females who died at younger ages (<80, 80–89) start declining earlier than those from the long-lived group (90+). They also start with larger values at age 40 and have a slower rate of increase with age at the interval 40–60 than the long-lived females (especially short-lived carriers of the e4 allele). The same is true for males (Figures 2(b) and 2(d)); short-lived males (especially noncarriers of the e4 allele) begin with larger values of CH at age 40 and their trajectories start declining earlier than those of the long-lived group (and the decline in the short-lived group is faster than in the long-lived group in noncarriers and, to a lesser extent, in carriers of the e4 allele). Figures 3(a) and 3(c) illustrate that the age trajectories of DBP for females who died at younger ages (<80, 80–89) also start declining earlier than those of the long-lived group (90+). Females in the short-lived group (<80) start with larger values at age 40 and then they have larger values at the interval 40–55 than the long-lived females (especially noncarriers of the e4 allele) followed by a faster decline afterwards, compared to the long-lived group. Trajectories for male noncarriers (Figure 3(b)) show patterns similar to those of female noncarriers: larger values of DBP at age 40, a higher level at ages 40–55 and then a faster decline at subsequent ages. Short-lived male carriers (Figure 3(d)) exhibit the same faster decline at advanced ages, compared to long-lived carriers, but the average values at younger ages are close in the two groups. 

The general conclusion from Figures 2 and 3 is that the average age trajectories of physiological variables in individuals dying at earlier ages markedly deviate from those of the long-lived groups and these patterns differ for carriers and noncarriers of the e4 allele. Long-lived individuals (90+ or 85+), compared to short-lived ones (<80 or <85), have consistently higher levels and a less steep decline of both CH and DBP at old ages (65+) when such levels naturally go down in aging human organism, which is in line with overall higher resistance to stresses in the former group. Various aging-related processes may jointly contribute to such differences. Application of the GenSPM allows for evaluating patterns of several such aging-related characteristics in carriers and noncarriers of the e4 allele.

### 3.2. Application of the Genetic Stochastic Process Model

**Figure 4:**
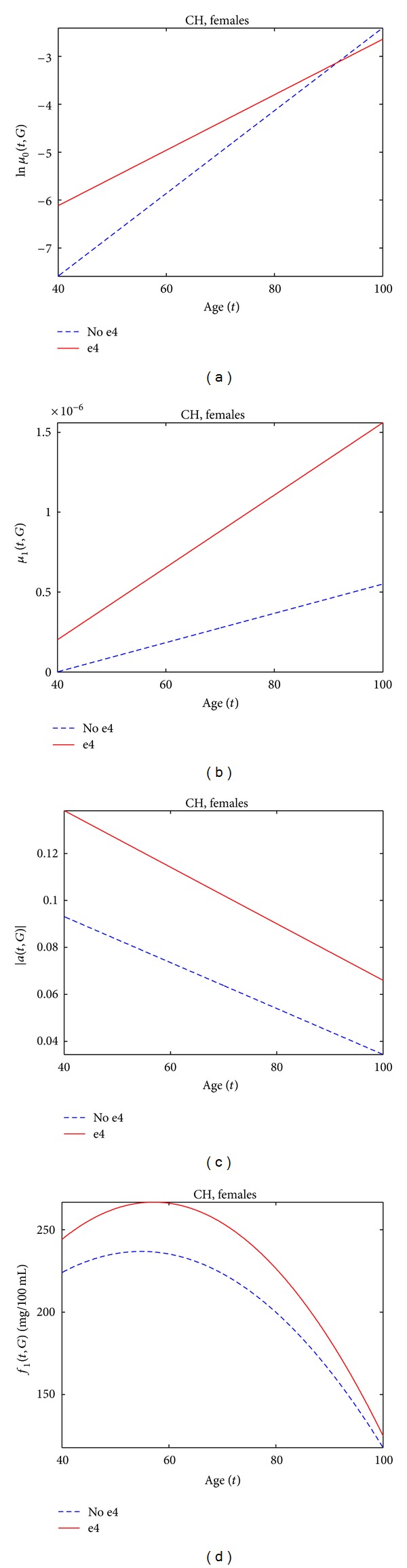
Application of the genetic stochastic process model to longitudinal measurements of total cholesterol (“CH”) and data on mortality for females in the Framingham Heart Study (original cohort). Estimates of the logarithm of the baseline hazard (a), the multiplier in the quadratic part of the hazard (b), the adaptive capacity (the absolute value of the feedback coefficient) (c), and the mean allostatic trajectory (d) for carriers (“e4”) and noncarriers (“No e4”) of the APOE e4 allele.

**Figure 5:**
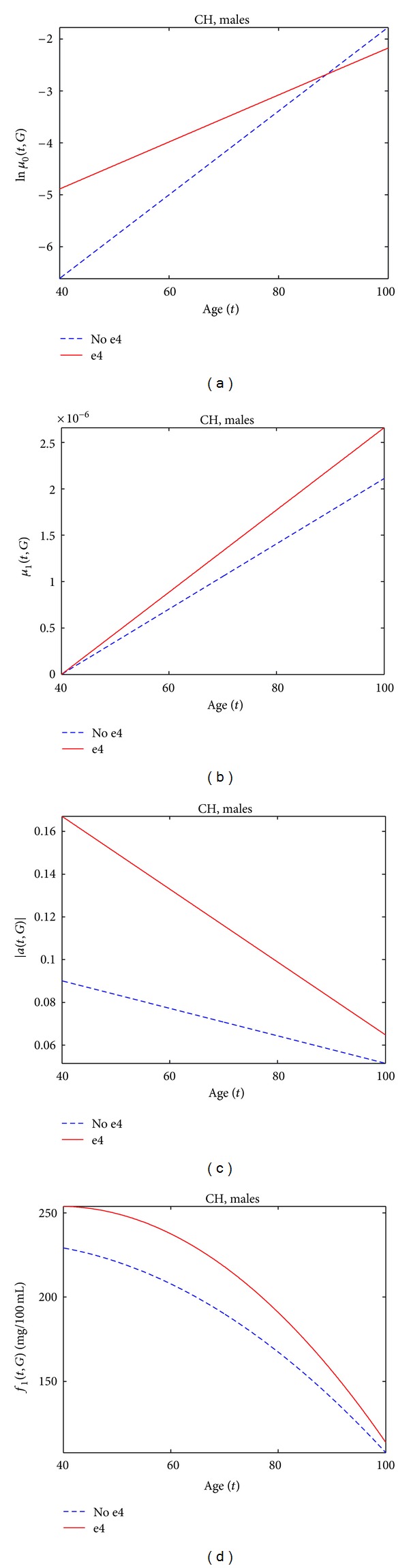
Application of the genetic stochastic process model to longitudinal measurements of total cholesterol (“CH”) and data on mortality for males in the Framingham Heart Study (original cohort). Estimates of the logarithm of the baseline hazard (a), the multiplier in the quadratic part of the hazard (b), the adaptive capacity (the absolute value of the feedback coefficient) (c), and the mean allostatic trajectory (d) for carriers (“e4”) and noncarriers (“No e4”) of the APOE e4 allele.

**Figure 6:**
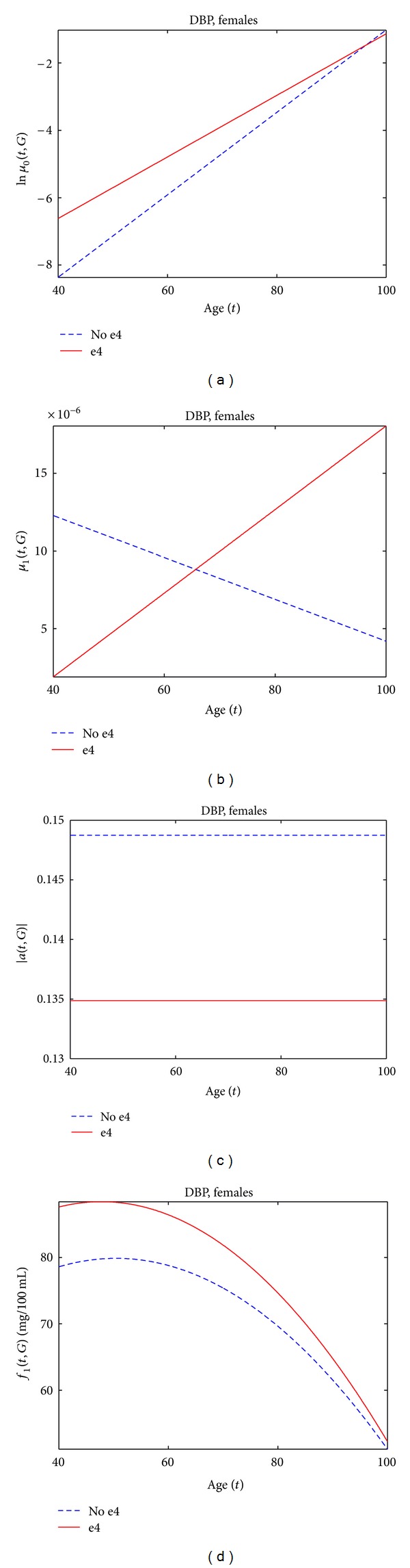
Application of the genetic stochastic process model to longitudinal measurements of diastolic blood pressure (“DBP”) and data on mortality for females in the Framingham Heart Study (original cohort). Estimates of the logarithm of the baseline hazard (a), the multiplier in the quadratic part of the hazard (b), the adaptive capacity (the absolute value of the feedback coefficient) (c), and the mean allostatic trajectory (d) for carriers (“e4”) and noncarriers (“No e4”) of the APOE e4 allele.

**Figure 7:**
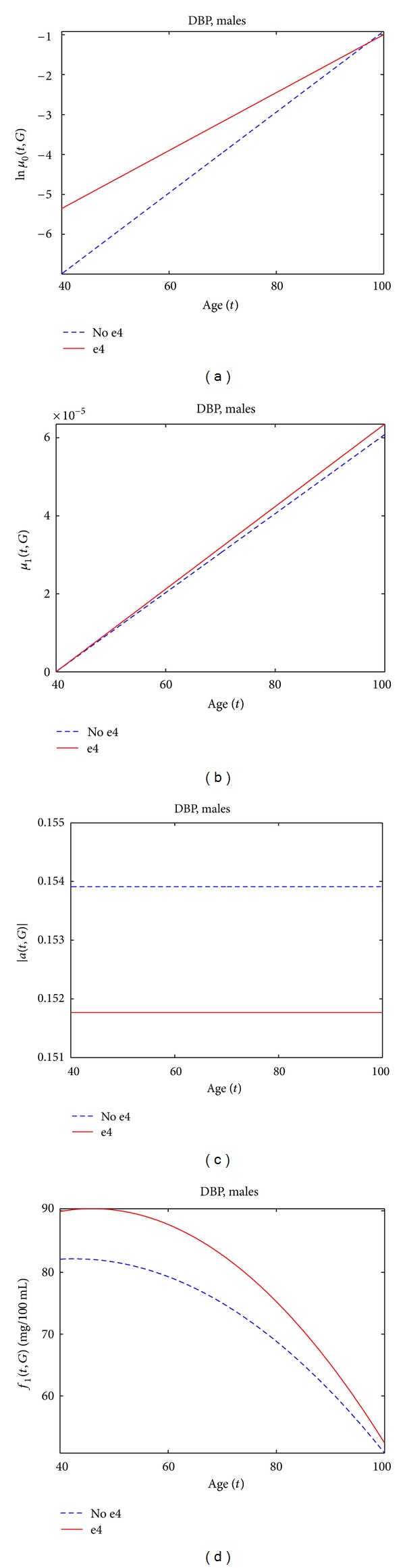
Application of the genetic stochastic process model to longitudinal measurements of diastolic blood pressure (“DBP”) and data on mortality for males in the Framingham Heart Study (original cohort): Estimates of the logarithm of the baseline hazard (a), the multiplier in the quadratic part of the hazard (b), the adaptive capacity (the absolute value of the feedback coefficient) (c), and the mean allostatic trajectory (d) for carriers (“e4”) and noncarriers (“No e4”) of the APOE e4 allele.

Estimates of parameters of the baseline hazard (*μ*_0_(*t*, *G*)), the multiplier in the quadratic part of the hazard (*μ*_1_(*t*, *G*)), the adaptive capacity *a*(*t*, *G*), the mean allostatic trajectory (*f*_1_(*t*, *G*)), and other parameters of the genetic stochastic process model applied to the FHS data on mortality and longitudinal measurements of physiological variables are given in Table 1. The table also contains information on testing various null hypotheses about coincidence of various components of the model (such as adaptive capacity, mean allostatic trajectory, etc.) in carriers and noncarriers of the e4 allele and other hypotheses on dynamic characteristics of the components of the model in the genetic groups (see “Note” after the table). Figures 4–7 display estimated components of the model (such as the logarithm of the baseline hazard, the multiplier in the quadratic part of the hazard, the adaptive capacity and the mean allostatic trajectory) for female and male carriers and noncarriers of the APOE e4 allele evaluated from data on CH and DBP.

The null hypotheses on the equality of baseline hazard rates in carriers and noncarriers of the e4 allele (column “ln⁡⁡*a*_*μ*_0__^*G*^” in Table 1) are rejected for both physiological variables and both sexes. Figures 4(a), 5(a), 6(a), and 7(a) illustrate the patterns of the logarithm of baseline hazard rates estimated for both physiological variables and both sexes. They show that noncarriers of the e4 allele have lower baseline rates at younger ages (i.e., smaller ln⁡*a*_*μ*_0__^*G*^) but they increase faster (i.e., they have larger *b*_*μ*_0__^*G*^) than the rates for carriers of the e4 allele resulting in the intersection of the rates at the oldest ages (around 90–100 years). This observation is in line with the findings in the literature that the effect of the e4 allele on survival diminishes with age [40] and the lack of association of APOE alleles with survival of centenarians [41].

The null hypotheses on zero quadratic part of the hazard (column “*a*_*μ*_1__^*G*^” in Table 1) are rejected in all cases for DBP but only for female carriers of e4 in case of CH. This suggests that deviations of DBP from the “optimal” trajectories results in a more substantial increase in the risk of death than in case of CH. This is evidenced also by Figures 4(b), 5(b), 6(b), and 7(b) showing larger values of the multiplier *μ*_1_(*t*, *G*) for DBP. Also the results indicate that there is no substantial difference in the patterns of *μ*_1_(*t*, *G*) between carriers and noncarriers (respective null hypotheses on *μ*_1_(*t*, 1) = *μ*_1_(*t*, 0) were not rejected in all cases).

The null hypotheses on age-independent U-shapes of the hazard (column “*b*_*μ*_1__^*G*^” in Table 1) are rejected for male carriers and noncarriers of the e4 allele in case of DBP. Respective estimates of parameter *b*_*μ*_1__^*G*^ are positive indicating the increase in *μ*_1_(*t*, *G*) with age for both carriers and noncarriers (Figure 7(b)). This corresponds to the narrowing of the U-shape of the mortality risk (as a function of DBP) with age. Hence, the “price” for the same magnitude of deviation from “optimal” values of DBP (in terms of an absolute increase in the mortality risk compared to the baseline level at that age) becomes higher for male carriers and noncarriers at older ages. This can be considered as a manifestation of the decline in resistance to stresses with age [12, 14] which is an important characteristic of the aging process [37, 38] leading to the development of aging-related diseases and death. It is important to note that our approach allows for indirect evaluation of this characteristic for carriers and noncarriers of the e4 allele in the absence of specific information on external disturbances (stresses) affecting individuals during their life course (such data are not available in the FHS). 

The results also revealed different age dynamics of the adaptive capacity in carriers and noncarriers of the e4 allele for different physiological variables. The null hypotheses on the equality of the adaptive capacity in carriers and noncarriers (column “*a*_*Y*_^*G*^” in Table 1) are rejected in all cases except DBP for males. Figures 4(c), 5(c), 6(c), and 7(c) show that in case of CH, carriers of the e4 allele have better adaptive capacity than noncarriers of this allele, whereas for DBP the opposite situation is observed. The age dynamics of the adaptive capacity is also different in case of CH and DBP. The null hypotheses on no aging-related decline in the adaptive capacity (column “*b*_*Y*_^*G*^” in Table 1) are rejected (at the 0.001 or 0.0001 level) in case of CH but there is no decline in the adaptive capacity for DBP (Figures 4(c), 5(c), 6(c), and 7(c)). These observations indicate that the mechanisms underlying the decline in the adaptive capacity in carriers and noncarriers may not work universally for all physiological indices. In case of CH, the decline in the adaptive capacity with age in both carriers and noncarriers of the e4 allele means that more time is needed for the trajectory of CH to approach the one that the organism tends to follow (i.e., the mean allostatic trajectory *f*_1_(*t*, *G*)) at older ages compared to younger ages. The decline in adaptive capacity is an important feature of aging [22–25] which may contribute to development of aging-related diseases and death. However, direct measurements of the adaptive capacity are typically lacking in available longitudinal studies of aging, health, and longevity. The use of the feedback coefficient in the equation for the age dynamics of a physiological variable in our model allows us to indirectly evaluate this from the data because the absolute value of this feedback coefficient characterizes the adaptive capacity [12–15]. 

The null hypotheses on the equality of the mean allostatic trajectories in carriers and noncarriers (column “*a*_*f*_1__^*G*^” in Table 1) are rejected (*P* < 0.0001) in all cases. This indicates that the processes regulating the age dynamics of physiological variables in carriers and noncarriers of the e4 allele force their age trajectories to follow different curves (which also do not coincide with the “optimal” trajectories). Figures 4(d), 5(d), 6(d), and 7(d) show that age trajectories of both CH and DBP in female and male carriers of the e4 allele are forced to larger values compared to noncarriers of this allele, although the difference between carriers and noncarriers diminishes at the oldest ages. 

## 4. Conclusions

To conclude, the major findings from the empirical analyses in this paper are the following.

- The long-lived female and male carriers and noncarriers of the e4 allele have different average age trajectories of CH and DBP (Figure 1).
- The average age trajectories of physiological variables (CH and DBP) in females and males dying at earlier ages markedly deviate from those of the long-lived groups. These patterns differ for carriers and noncarriers of the e4 allele (and also by sex). Long-lived individuals have consistently higher levels and a less steep decline of both CH and DBP at old ages compared to short-lived individuals (Figures 2 and 3).

Application of the GenSPM revealed different patterns of regularities in aging-related characteristics (adaptive capacity, decline in stress resistance, mean allostatic trajectories, and the baseline hazard rate) in carriers and noncarriers of the APOE e4 allele. Such aging-related characteristics cannot be calculated directly from the longitudinal data because of the lack of respective measurements. 

The major findings in applications of the GenSPM are the following.

- Noncarriers of the e4 allele have lower baseline mortality rates at younger ages but they increase faster than the rates for carriers of the e4 allele resulting in the intersection of the rates at the oldest ages (Figures 4(a), 5(a), 6(a), and 7(a) and column “ln⁡*a*_*μ*_0__^*G*^” in Table 1).
- Deviations of DBP from the “optimal” trajectories results in a more substantial increase in the risk of death than in case of CH in both carriers and noncarriers of the e4 allele (Figures 4(b), 5(b), 6(b), and 7(b) and column “*a*_*μ*_1__^*G*^” in Table 1).
- We found that the U-shape of the mortality risk as a function of DBP narrows with age in male carriers and noncarriers of the e4 allele (Figure 7(b) and column “*b*_*μ*_1__^*G*^” in Table 1) which can be considered as a manifestation of the decline in resistance to stresses with age.
- The pattern of the adaptive capacity is different in case of CH and DBP. In case of CH, carriers of the e4 allele have better adaptive capacity than noncarriers of this allele whereas for DBP the opposite situation is observed (Figures 4(c), 5(c), 6(c), and 7(c) and column “*a*_*Y*_^*G*^” in Table 1).
- The age dynamics of the adaptive capacity is also different for CH and DBP. The decline is significant in case of CH but there is no decline for DBP (Figures 4(c), 5(c), 6(c), and 7(c) and column “*b*_*Y*_^*G*^” in Table 1).
- The “mean allostatic trajectories” of CH and DBP are different in carriers and noncarriers of the e4 allele (larger values in carriers compared to noncarriers ) and they are different from the “optimal” trajectories minimizing the risk of death (Figures 4(d), 5(d), 6(d), and 7(d) and column “*a*_*f*_1__^*G*^” in Table 1).

These differential patterns of aging-related characteristics may contribute to differences between the shapes of survival functions and average age trajectories of respective physiological variables in carriers and noncarriers of the e4 allele as well as between females and males. The underlying determinants of such differences in aging-related characteristics require additional studies. Taking into account the possibility of tradeoffs in the effects of the APOE polymorphism on the ages at onset of aging-related diseases [5], it is important to consider applications of the model to data on incidence of such diseases (e.g., cancer and CVD) and cause-specific mortality. Such applications may reveal trade-offs in the effects of the APOE polymorphism on regularities of different aging-related characteristics which may be masked in the analyses of such a complex phenotype as survival or mortality from all causes combined. 

## Figures and Tables

**Table 1:** Estimates of parameters of the genetic stochastic process model applied to data on mortality and longitudinal measurements of total cholesterol (“CH”) and diastolic blood pressure (“DBP”) in female (“F”) and male (“M”) carriers (“e4”) and non-carriers (“no e4”) of the APOE e4 allele in the Framingham Heart Study (original cohort).

Variable	Sex	Allele	Baseline Hazard (μ_0_(*t*, *G*))			Multiplier in quadratic part of hazard (μ_1_(*t*, *G*))		Adaptive capacity (*a*(*t*, *G*))		Mean allostatic trajectory (*f*_1_(*t*, *G*))			Other parameters			ln *L*
			ln⁡ *a*_μ_0__^*G*^	*b*_μ_0__^*G*^	σ_2_^*G*^	*a*_μ_1__^*G*^	*b*_μ_1__^*G*^	*a*_*Y*_^*G*^	*b*_*Y*_^*G*^	*a*_*f*_1__^*G*^	*b*_*f*_1__^*G*^	*c*_*f*_1__^*G*^	σ_0_^*G*^	σ_1_^*G*^	*p*_1_	
CH	F	no e4	−7.59^#^	0.086	0.00	−0.0037	0.0009	−0.093^†^	0.978^†^	223.99^†^	1.731	−0.0583	39.05	14.30	0.645	−171761.430
		e4	−6.12	0.058	0.00	−0.0071^#^	0.0023	−0.138	1.204^§^	244.08	2.645	−0.0772	49.14	22.21		
CH	M	no e4	−6.61^†^	0.081	0.00	−0.0141	0.0035	−0.090^†^	0.644^§^	229.18^†^	−0.588	−0.0239	38.19	13.60	0.666	−127202.469
		e4	−4.89	0.045	0.06	−0.0177	0.0044	−0.167	1.705^†^	253.78	−0.055	−0.0379	48.24	22.30		
DBP	F	no e4	−8.37^†^	0.122	0.00	0.1767^#^	−0.0135	−0.149^#^	0.000	78.60^†^	0.245	−0.0117	8.96	4.96	0.634	−151149.750
		e4	−6.62	0.091	0.00	−0.0886^#^	0.0269	−0.135	0.000	87.62	0.207	−0.0133	14.69	6.44		
DBP	M	no e4	−6.99^†^	0.101	0.00	−0.4053^#^	0.1013^#^	−0.154	0.000	82.15^†^	0.050	−0.0096	8.53	4.86	0.640	−109616.899
		e4	−5.35	0.073	0.00	−0.4233^§^	0.1058*	−0.152	0.000	89.87	0.156	−0.0130	13.53	6.55		

Notes:

(1) ln *L* logarithm of the likelihood function.

(2) The estimates of some parameters are rescaled for better visibility in the table: *a*_μ_1__^*G*^are multiplied by 10^4^; *b*_μ_1__^*G*^ are multiplied by 10^5^; *b*_*Y*_^*G*^ are multiplied by 10^3^.

(3) The symbols after the numbers in the following columns of Table 1 denote *P* values (evaluated by the likelihood ratio test) for different null hypotheses.

Column “ln  *a*_μ_0__^*G*^”: null hypothesis—baseline hazard rates coincide in carriers and non-carriers of the e4 allele, that is, μ_0_(*t*, no *e*4) = μ_0_(*t*, *e*4) (respective symbols are shown in rows “no e4”).

Column “*a*_μ_1__^*G*^”: null hypothesis—zero quadratic part of the hazard (separately for carriers and non-carriers), that is, μ_1_(*t*, no *e*4) = 0 for rows “no e4”, μ_1_(*t*, *e*4) = 0 for rows “e4”.

Column “*b*_μ_1__^*G*^”: null hypothesis—age-independent U shapes of the hazard (separately for carriers and non-carriers), that is, *b*_μ_1__^1^ = 0 for rows “no *e*4”, *b*_μ_1__^0^ = 0 for rows “e4”.

Column “*a*_*Y*_^*G*^”: null hypothesis—adaptive capacities coincide in carriers and non-carriers, that is, *a*(*t*, no *e*4) = *a*(*t*, *e*4) (respective symbols are shown in rows “no e4”).

Column “*b*_*Y*_^*G*^”: null hypothesis—no aging-related decline in the adaptive capacity (separately for carriers and non-carriers), *b*_*Y*_^1^ = 0 for rows “no e4”, *b*_*Y*_^0^ = 0 for rows “e4”;

Column “*a*_*f*_1__^*G*^”: null hypothesis—“mean allostatic trajectories” coincide in carriers and non-carriers, that is, *f*_1_(*t*, no *e*4) = *f*_1_(*t*, *e*4) (respective symbols are shown in rows “no e4”).

The symbols in these columns denote: ^†^ *P* < 0.0001; ^§^0.0001 ≤ *P* < 0.001; ^#^0.001 ≤ *P* < 0.01; *0.01 ≤ *P* < 0.05, for respective null hypotheses. The absence of symbols after the numbers in these columns means that respective *P*-values exceed 0.05. Note that all other columns in the table, except the columns mentioned above, are not used to represent information on testing any null hypotheses and therefore they do not contain any symbols.
